# And sympathy is what we need my friend—Polite requests improve negotiation results

**DOI:** 10.1371/journal.pone.0212306

**Published:** 2019-03-13

**Authors:** Yossi Maaravi, Orly Idan, Guy Hochman

**Affiliations:** 1 The Adelson School of Entrepreneurship, Interdisciplinary Center (IDC), Herzliya, Israel; 2 Baruch Ivcher School of Psychology, Interdisciplinary Center (IDC), Herzliya, Israel; University of Amsterdam, NETHERLANDS

## Abstract

The wording negotiators use shapes the emotions of their counterparts. These emotions, in turn, influence their counterparts’ economic decisions. Building on this rationale, we examined how the language used during negotiation affects discount rate and willingness to engage in future deals. In three studies, participants assumed the role of retailers. Alleged counterparts (actually a computerized program) asked for a discount under three conditions: request, want, and demand. Results show that less extreme language (request/want) resulted in better outcomes than demanding a discount. Moreover, while the language used by the customer had an effect on experienced emotions, the positive emotions (sympathy and empathy) participants felt toward the customer mediated the relationship between the linguistic cue and the negotiation outcome. Our results inform both psycholinguistic research and negotiation research by demonstrating the causal role of linguistic cues in activating concept-knowledge relevant to different emotional experiences, and point to the down-the-line impact on shaping negotiation preferences.

## Introduction

“*He who wants to persuade should put his trust not in the right argument*, *but in the right word*.*”*

(Joseph Conrad)

Negotiation is an important social phenomenon, for we all negotiate on a day-to-day basis with our business partners, colleagues, friends and even family members. In a typical negotiation, one party makes the first offer, his or her counterpart responds, and from there on the negotiation evolves until it reaches an agreement or an impasse [1]. In distributive negotiations, in which the process is competitive and the gain of one negotiator is the loss of the other, the rate of discount is of significance to both parties [2]. Additionally, a negotiation situation could be one incident in an established long-term relationship. Thus, discount rate and the continuation of the relationship, which consequently influences the willingness to engage in future deals, are important aspects in negotiation outcomes [3,4]. Previous research has focused on different dimensions of the negotiation process which may lead to different outcomes, such as the first offer and the way it affects the counteroffer (e.g. [5]), alternatives within the negotiation process [6], cultural differences [7], politeness [8], and reference points, such as the current market data [9]. The current research focuses on the role of language in the context of negotiations, acknowledging its potential as a tool for promoting successful negotiation outcomes.

Psycholinguistic research on the effect of language suggests that language has the power to shape emotions [10] within a persuasion framework [11] as well as shaping negotiating preferences [12,13]. Building on this line of research, in the current work we explored a subtle means of influence in the context of negotiations. Specifically, we examined whether linguistic differences in asking for a discount (request, want, or demand) in the context of negotiations would induce different levels of positive (sympathy, empathy) or negative (anger) emotions and in turn, different outcomes (discount rate, willingness to engage in future deals). Importantly, in the current study we do not employ a full interactive process. Rather, we aim to examine how language severity affects the emotions that people experience towards their counterparts and how these experienced emotions effect important negotiation outcomes (e.g., willingness to engage in future negotiations). To enable such an examination, we use a non-interactive situation and focus on the first steps of the negotiation process, namely the response to a first offer [14, 15]. This enables us to isolate the direct effect of language extremity, and prevent the influence of other factors (which we do not control for).

### Language in negotiations

Attempts to understand the role of linguistic style in negotiation, specifically hostage negotiations, revealed that successful negotiations were associated with turn taking, reciprocation of positive affect, a focus on the present rather than the past, and a focus on alternatives rather than on competition [16]. Furthermore, procedural framing within the negotiation process was examined and found to have an impact on the negotiation outcome, in that negotiators were more concession averse and claimed higher individual value when negotiation proposals were framed to accentuate their own rather than their counterpart’s resources [17].

Procedural framing [17] and communication are part of the negotiation process and negotiators are required to make language choices that highlight or weaken aspects of, or attitudes towards, the issue that is on the table [18]. These linguistic choices constitute the negotiator’s orientation regarding the relationship with the negotiating party [19, 20]. For example, in our context, requesting creates a relational context of moving towards/with, as expressed by the positive emotions, which in turn affects the measured negotiation outcomes. Since requesting a discount can be viewed as an attempt to influence the negotiating party, the current research adopts a persuasion framework, answering Malhotra and Bazerman’s [21] call for research on psychological influence in negotiation. The fact that different use of linguistic cues may result in different levels of emotions, attitude judgments and behavior may suggest that negotiation research can indeed benefit from exploring linguistic cues within a persuasion framework.

### Linguistic cues in persuasion

Individuals, who receive a message meant to persuade them, are attentive to various aspects of the message (e.g. [22, 23]). One such aspect may be the linguistic cues provided by the senders of these messages. Linguistic cues may be judged not only by what they communicate, but also by how they are communicated [24]. People can intentionally or unintentionally employ a linguistic style that perceivers/receivers use in forming impressions and attitudes [25]. One’s linguistic style can be so central that it not only affects the persuasiveness of an appeal, but may also be considered a defining feature of the person presenting the appeal. More specifically, the language style may not necessarily change the content of information, but can influence both the perceptions of the communicator and the message [26]. Much like other variables in a persuasion context (e.g., mood, emotions), linguistic styles can serve multiple roles, influencing judgments directly or serving to bias effortful thinking about the attitude object [23].

One particular style associated with valence is the extremity of the language used. Language extremity was found to affect message processing and behavioral intentions [11]. Linguistic extremity has been considered an important and complex characteristic in the persuasion setting because it is associated with increases [27] and decreases [28] in credibility, and in message discrepancy [29]. In the context of negotiation, linguistic extremity increases counter-arguing [30], and resistance to persuasion [31]. Individuals manipulate or vary their speech in order to achieve a particular outcome [32], on both the cognitive and emotional levels. Given that language extremity may relate to resistance to persuasion, we hypothesized that *the extremity of the language would be negatively correlated with discount rate (H1a) and willingness to engage in future deals (H1b)*. In a pretest (as outlined in the results of Study 1 and 2), we show that the words we used represent different levels of language extremity. Specifically, “demand” was perceived as presenting the highest level of extremity, while”request” was perceived as presenting the lowest level of extremity.

Indeed, the fact that language extremity should influence negotiation outcome might not be surprising and novel. However, the main contribution of the current work is not in demonstrating the impact of language on behavior. Rather, we aim to examine the impact of linguistic cues on emotions, which serve as a mediator between the cue and the outcome of the negotiation (see hypotheses 3 and 4 as follows).

### Language, emotions and perceptions

In 1956, Whorf [33] proposed that language influences our thinking and consciousness. Following Whorf’s proposal, the underlying question that has interested psycholinguists in the past decades is to what extent does language carve our perception of reality and our emotional experiences? Evidence from social, cognitive, neuropsychological, and cross-cultural studies is consistent with the psychological constructionist view that language helps constitute emotion [10]. It allows people to acquire, organize, and use the concept knowledge that is an essential element in emotion perceptions [34,10]. Distinct linguistic forms automatically activate social meaning independent of their explicit semantic content [35,36]. When social meaning associated with a specific linguistic form overlaps with appraisals underlying specific emotions, the linguistic cue can evoke the corresponding emotion [10].

Research shows that even the mere use of a distinct grammatical category (e.g. adjective versus noun form) can activate different construal, affecting judgment and behavioral preferences [37,38,39]. For example, results across three experimental studies, in the context of the Israeli-Palestinian conflict supported this conclusion, with reduction in anger mediating the salutary impact of noun versus verb labels on support for conciliatory policies [40]. Acknowledging the importance of emotions in affecting judgment and activating perceptions, the current research examines the role of language in inducing emotions in the context of the negotiation process and outcomes. Given the central role of language in overlapping with appraisals underlying specific emotions, we hypothesized that *language extremity would be negatively correlated with positive emotions and positively correlated with negative emotions (H2)*.

### Emotions and valence in negotiations

Emotions play a fundamental role in shaping negotiations [41,42,43] and are thus considered a natural component within the study of negotiations [44]. Studies have shown the detrimental effect of emotions in negotiations [45], alongside the complexity of affect in shaping the negotiation process and outcomes [43]. Empirical research has revealed the impact emotions have on the negotiators’ behavior and decisions during the negotiation process, and as a result on the outcomes [46].

From a standard economic perspective, one may conclude that emotions and cues that do not convey economic consequences, such as the exact wording of otherwise identical requests, should not influence negotiators [47]. However, research in both persuasion and behavioral decision-making has long established that emotions do in fact influence decision makers. One prime example is the Ultimatum Game [48]. In this bilateral bargaining game, one player (the proposer) must decide how to split a certain amount of money (the pie. e.g., 20$) between himself and another player. The second player (the responder) has to decide whether she wants to accept the proposed split, in which case both players earn the amount proposed, or reject it, in which case both earn nothing. In these settings, the standard economic model suggests that proposers should offer the smallest possible amount (e.g., $1), and responders should accept it. However, the typical results suggest that proposers tend to offer a fair share of 40–50% of the pie, and that the majority of respondents reject unfair (below 30%) offers (e.g., [48]; for a review, see [49]). These results and the results of other related experiments (e.g., [50,51]) are attributed not to economic, but rather to psychological, and more specifically, emotional valence, related to equity or fairness [52].

While the Ultimatum Game mainly focuses on emotional reactions around fairness, over the past two decades abundant research has also established the centrality of other emotions in negotiation processes [53, 54]. For example, Kopelman, Rosette and Thompson [55] have demonstrated how strategically displaying positive, negative, or neutral emotions affected negotiation outcomes. In one of their experiments, which is also in-line with the results of the current research, negotiators who displayed positive—but not negative or neutral—emotions in face-to-face dispute simulation, increased the likelihood of future business relationship.

Drawing on the role that emotions play in negotiations and the dynamic interpersonal setting involved in the negotiation process [41,42,43], the current research focuses on positive and negative emotions that are evoked by the language used during the negotiation. This language, in turn, affects the processing of the message, and can thus make it more or less favorable. As a result, the language used may affect two significant negotiation outcomes—the given discount rate and the willingness to engage in future negotiations. Positive emotions can foster cooperativeness and trustworthiness, concession from others [56], as well as problem solving approach and integrative agreements [57]. In addition to obtaining a better substantive outcome, positive affect has been found to increase the affective and relational satisfaction of the parties in a negotiation [58]. The display of positive affect encourages the continuation of long-term business relationships and increases the chance that one party will perceive the other party as fair and cooperative [55]. This, in turn may increase the level of trust and enhance potential mutual gains for both sides of the negotiation.

Research suggests that negative emotions have a stronger effect on behavior than positive emotions (see e.g., [59,60]). Negative emotions may portray a negotiator as aggressive and competitive, and may lead to negative consequences for the non-complying opponent [56]. Contrary to positive affect, negative affect has been shown to decrease initial offers, achieve fewer mutual gains and decrease the aspiration to negotiate in the future [43].

Griessmair [61] reveals that differences in negotiation outcomes are largely due to whether the negotiators experience a negative or positive emotional climate. The impasse dyads experience a negative emotional climate, whereas those that reach a satisfactory settlement experience positive emotions. Furthermore, Griessmair’s findings point to differences between emotions that address individual goal realization as opposed to emotions that focus on relational interpersonal aspects of negotiations. The latter study focuses on emotional expressions following a proposed offer and their influence on the dynamics and development of the emotional experiences during the negotiation. In the current study, we focus on the emotions evoked following a request or demand to receive a discount. The emphasis is not on the actual request, but rather on the linguistic cue conveying the extremity of the request. Relying on research on distinct emotions [43,45], and emotional valence [13,59] within the negotiation process, the current studies examined the levels of induction of sympathy, empathy (positive valence) and anger (negative valence).

### Sympathy, empathy and anger in negotiations

Drawing on research on the influence of specific emotions on negotiation-related cognition and behavior [42,61,62], the current research focuses on sympathy, empathy and anger in an attempt to understand the role of emotional valence within the negotiation process. While anger is clearly an emotion [63,64,65], it is important to clarify that both sympathy and empathy, as discussed and defined in detail below, are also defined in social psychology [66] and social-cognitive neuroscience [67] as feelings or emotions [68] More specifically, sympathy and empathy–much like embarrassment, pride or schadenfreude—are considered "social emotions": emotions that depend on interacting with other people and are central for promoting or discouraging socially appropriate or inappropriate behaviors [69].

In general, sympathy and anger have been found to differ in the source that evokes them. Specifically, when the source is a controllable cause (e.g., a client did not hear the shop owner’s response because he was not paying attention), anger is the typical emotional response. However, when the source is an uncontrollable cause (e.g., the client did not hear the shop owner’s response due to noisy renovations at the store), sympathy is more likely to be evoked [70]. Furthermore, anger has been associated with retaliation and aggressive action, and with distancing oneself from the source of anger. It stems from the perception that others are carrying out an action that is unjust, unfair, or contrary to acceptable societal norms [63]. Specifically, anger has been shown to have a negative impact on negotiations [64] and may be triggered by violating fairness, concession making or trust and by presenting excessive demands [65]. Given the central role of anger in shaping adversarial policy preferences, we hypothesized that *anger would mediate the effect of language extremity on discount rate (H3a) and the willingness to negotiate in the future (H3b)*.

Sympathy [71] and empathy [72] have both been found to positively enhance the negotiation process, and yet each one works in different ways. Sympathy is an altruistic emotion that results from perceiving the other side as vulnerable (due to an uncontrollable cause) [73]. As such, sympathy often motivates individuals toward acts that are aimed at improving or altering the vulnerable state. Research investigating the use of appeals to sympathy in the negotiation process found that an active appeal to the counterpart’s sympathy improved distributive and integrative negotiation outcomes. The elicitation of sympathy was more effective than rationality and fairness, especially among low power negotiators [71].

Empathy includes knowing the other’s thoughts and feelings, taking her/his perspective [74], and in particular, how one thinks events affect the experiences of the other [75]. The latter, relates to the perceived emotional state that observers encounter in others [76], in particular, evoking such affective responses as sympathy, compassion, and tenderness [77]. Empathy, may however lead people to violate norms of equity and equality and provide preferential treatments [78]. Examining issues "through the eyes" of the opposite party may also lead to favoring the other side’s interest and potentially lose gains.

Previous research showed that empathy could be beneficial in situations that relied on an affective understanding of and connection with others [62]. These findings demonstrated that understanding the interests of opponents in decision-making interactions is apparently more valuable than having an emotional connection [79]. Furthermore, as suggested by Galinsky and his colleagues [79], regarding negotiation benefits that may emerge over time, empathy may facilitate a long-term process towards an agreement in that it increases levels of satisfaction during the negotiation process. Given the role of sympathy in enhancing the negotiation process and the role of empathy in facilitating long-term negotiation process, we hypothesized that *sympathy and empathy would mediate the effect of language extremity on discount rate (H4a) and willingness to negotiate in the future (H4b)*.

## Study 1

### Materials and methods

#### Ethics statement

The study and consent procedure were reviewed and approved by the Interdisciplinary Center’s School of Psychology Institutional Review Board before it was administered. In accordance with the ethics protocol, participants provided electronic consent for participation at the beginning of the study.

#### Participants

One-hundred twenty-five undergraduate students (41 male) from a private university in the center of Israel participated in the experiment. All participants were native Hebrew speakers, who attended a course on language and thought. Thus, sample size was not determined a-priori. The average age was 25.0 years (SD = 2.2). Participation was voluntary and conducted upon consent at the end of class. Participants were randomly assigned to one of three between-subject conditions.

#### Design and procedure

Participants read a web-based negotiation vignette in which they were asked to assume the role of an air-conditioner seller (for a full version of the questionnaire in English, and all data see https://osf.io/8bzvk/). All vignettes were in Hebrew. The vignette informed participants that while each unit had a catalog price that was presented to prospective customers; they could sometimes give customers a discount. Then, participants were told that a customer asked for a quote on a split-unit air-conditioner, so they provided the customer with the unit’s catalog price, which was 4000 NIS (approximately 1,100 USD). In return, the customer responded by asking for a discount from the catalog price, which was manipulated in three between-subject conditions. The customer’s response was the only aspect of the vignette that varied across conditions. This manipulation was done to examine the specific effect of the linguistic cue on the participants’ decision and experienced emotions. In the *request* condition, the response of the customer translated “May I request a discount for the air conditioner?”, while in the *want* condition the response was “I want a discount for the air conditioner!” Finally, in the *demand* condition, the response was “I demand a discount for the air-conditioner!”

Before responding to the customer and deciding on a discount, participants in all conditions were required to answer questions that were aimed at examining how they felt about the response of the customer. Specifically, participants were required to indicate, “How angry are you at the customer”, “How much empathy do you feel for the customer”, and “How much sympathy do you feel for the customer”. Participants were required to indicate their level of agreement with the items on a 1–6 Likert-scale (1- not at all, 6 –extremely). To mask the true aim of the study, several filler questions were included (e.g., participants were also asked “to what extent are you disgusted by the customer” on the same 1–6 Likert-scale). For the complete set of questions included in all three studies, see https://osf.io/8bzvk/

After the rating of emotions, participants were required to indicate the amount of discount (in percentage) they would agree to give the customer (between 0 and 100%). Once the participants recorded their answers, they were informed that the customer responded with “The discount is acceptable. I will buy the item at the catalog price, minus the discount you gave me” and the deal was made. Following the deal, the participants were required to indicate (on the same 1-6-point scale) “to what extent they wish to conduct business with this customer in the future,” and “the extent in which they are satisfied with the price they got for the unit”.

The experiment was conducted in a classroom. A research assistant wrote the link to the survey on the board, and participants logged in via their personal electronic devices (laptops, smartphones etc.). Participants were not given a time limit.

In addition to the main study, a separate pretest (n = 77) was conducted to verify whether the words ‘request’, ‘want’, or ‘demand’ represent different levels of language extremity. Participants were undergraduate students from the same private university who attended a different course. All participants were native Hebrew speakers. They were asked to indicate to what extent, ‘I request, ‘I want’, and ‘I demand’ (in Hebrew, presented at a random order) represented a soft or tough appeal, on a 10-point scale (1 = very soft, and 10 = very tough).

### Results and discussion

#### Manipulation check

The average rating of soft-tough was highest for ‘I demand’ (Mean = 8.95, SD = 1.79), moderate for ‘I want (Mean = 4.79, SD = 1.79), and lowest for ‘I request’ (Mean = 3.56, SD = 2.28). Repeated-measures ANOVA revealed that the language used had a significant effect on participants’ ratings (F(2, 152) = 148.426, p < 0.0001, η_p_^2^ = .66). In addition, planned contrast further revealed a significant difference between ask and want (t(76) = -3.791, p < 0.0001), want and demand (t(76) = -15.89, p < 0.0001), and ask and demand (t(76) = -14.01, p < 0.0001). This pattern of results supports our claim that the language used affects perceived language extremity, and that "demand" leads to the highest level, while "request" leads to the lowest level of extremity.

#### Discount rate

**Fig 1:**
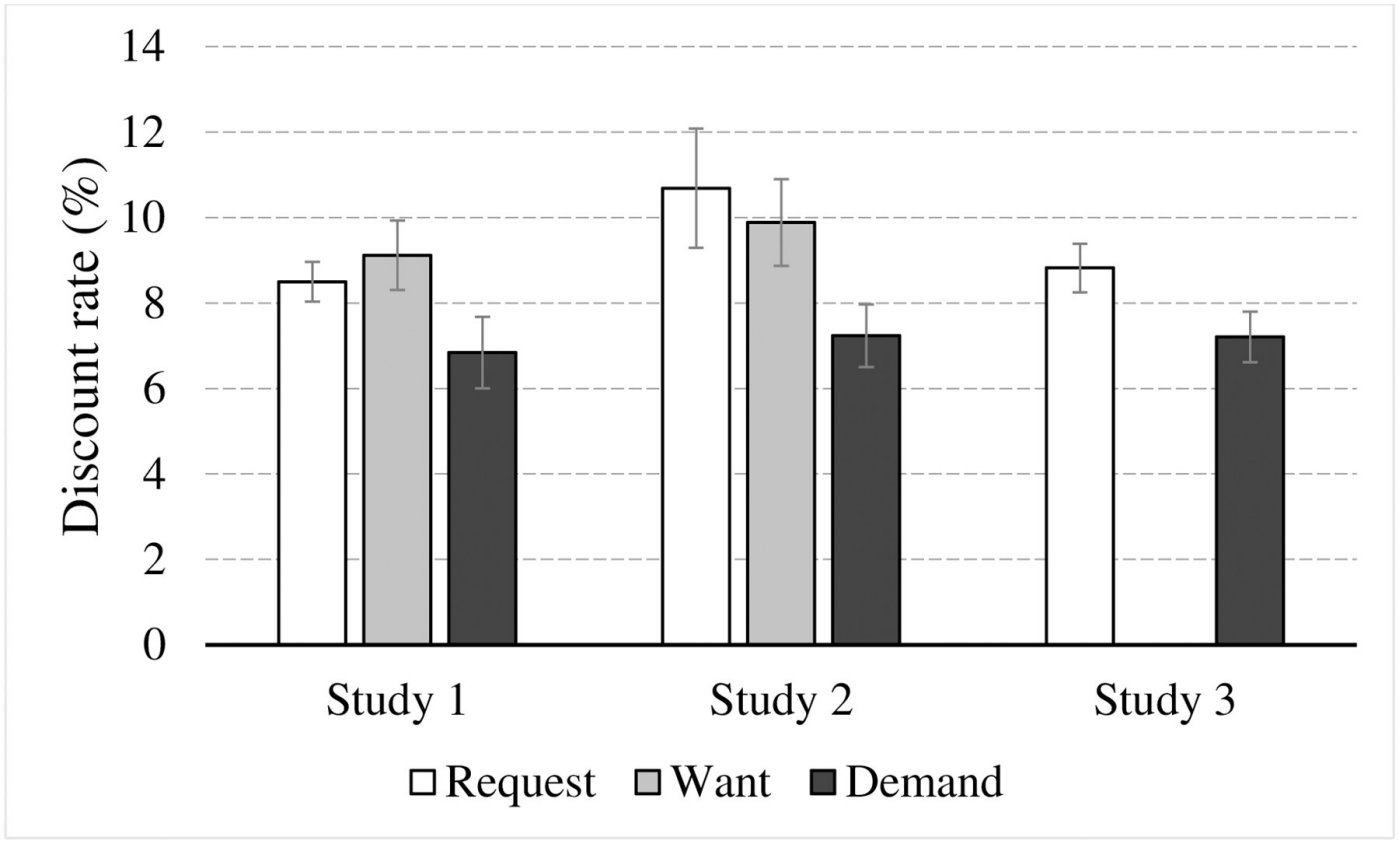
Averaged discount rate as a function of the language used to request a discount in all three experiments. Vertical lines depict standard error.

To test H1a we conducted ANOVA with planned contrast to examine the effect of language extremity on discount rate. The descriptive statistics for the analysis are presented in Fig 1 (left columns). As we predicted, language extremity had a marginally significant effect on discount rate (F(2, 122) = 2.614, p = 0.08, η_p_^2^ = .041). As can be seen in Fig 1, the discount rate was higher for requesting and wanting (Mean = 8.5%, SD = 3.0, 95% CL [7.05, 9.95], and Mean = 9.1%, SD = 5.3, 95% CL [7.70, 10.50], respectively) than for demanding a discount (Mean = 6.8%, SD = 5.3, 95% CL [5.40, 8.29]). Planned contrast further revealed that discount rate was significantly different between want and demand (p < 0.05), and marginally significant between request and demand (p = 0.1). No difference was found between request and want (p = 0.55). This pattern of results supports H1a.

Next, we tested H2, H3a, and H4a. Since our manipulation check results suggested that the language used falls on an extremity of language continuum, we conducted mediation analysis using the PROCESS macro (PROCESS Model 4; [80]) in SPSS v21 with bias-corrected bootstrapped confidence intervals (CI). The results revealed that language extremity (as was used in the ANOVA) was negatively related to empathy (β = -.463, SE = 0.13, 95% CL [-.717, -.209], p < 0.0001; R^2^_Adj_ = 9.6%) and sympathy (β = -.55, SE = 0.12, 95% CL [-.782, -.316], p < 0.0001; R^2^_Adj_ = 15.0%), and positively correlated with anger (β = .63, SE = 0.12, 95% CL [.391, .878], p < 0.0001; R^2^_Adj_ = 17.8%). Thus, in support of H2, language extremity was negatively correlated with positive emotions and positively correlated with negative emotions.

**Fig 2:**
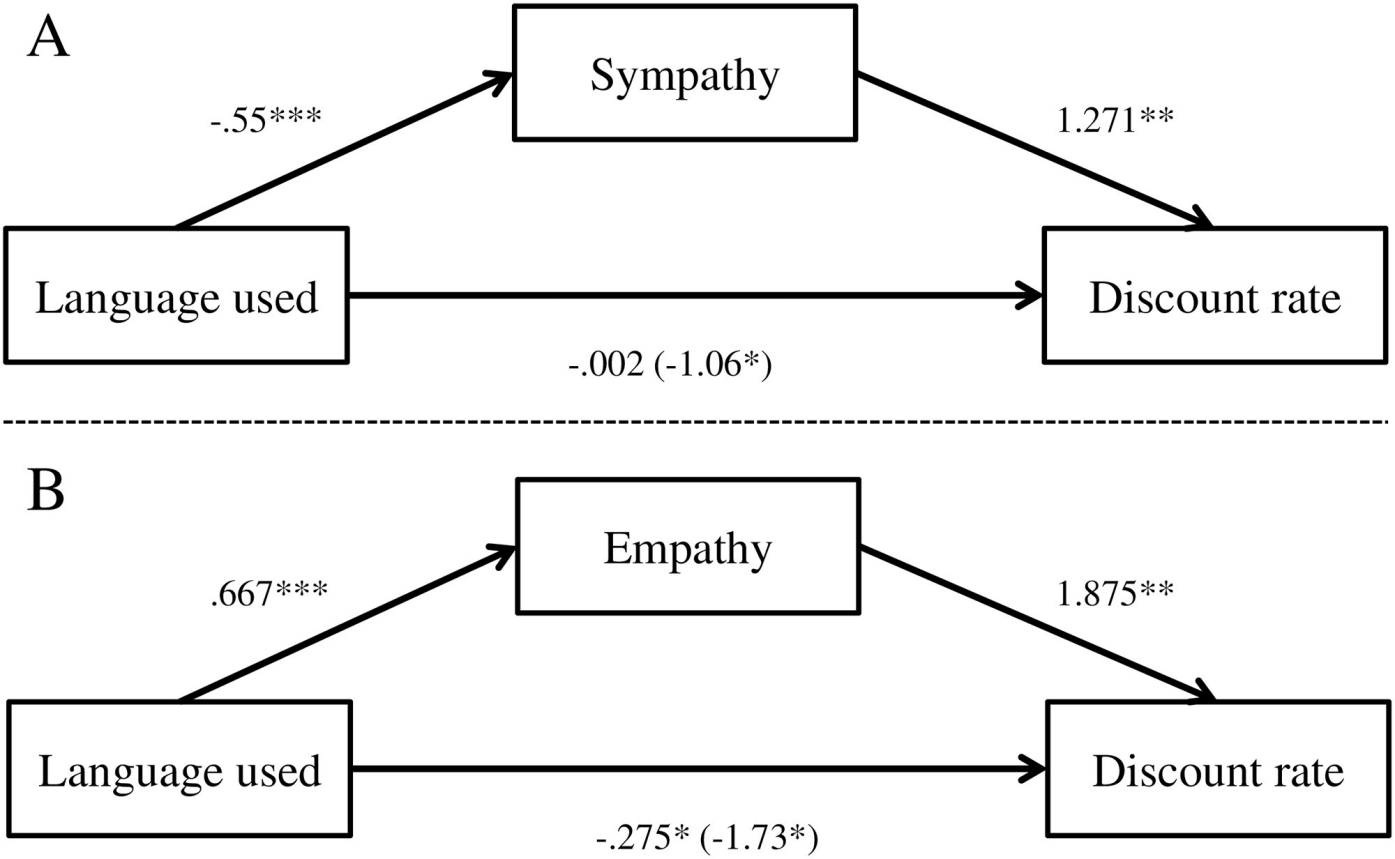
The effect of the language used on discount rate mediated by experienced emotions. All entries are raw (unstandardized) coefficients. The association between the mediator and the DV is represented by a coefficient from a model where the IV is also a predictor of the DV. Numbers in parentheses refer to the direct effect of the language used on discount rate. Panel A: Study 1. Panel B: Study 2. * < 0.05, ** < 0.01, *** < 0.0001.

In addition, in line with the ANOVA results, language extremity was negatively related to discount rate (β = -1.06, SE = 0.51, 95% CL [-2.073, -.052], p < 0.05; R^2^_Adj_ = 9.2%). However, when empathy, sympathy and anger were entered to the model as mediators, language extremity no longer had a significant relationship with discount rate (β = -.002, SE = 0.56, 95% CL [-1.11, -1.105], p = 0.97), and only sympathy (β = 1.271 SE = 0.45, 95% CL [.39, 2.153], p < 0.01) remained significant (F(5, 119) = 6.121, p < 0.0001; R^2^_Adj_ = 20.5%). Thus, H3a was not supported. However, the pattern of results is consistent with a mediation effect (H4a) for sympathy alone. The mediation results are illustrated in Fig 2 (Panel A).

#### Future deals

**Fig 3:**
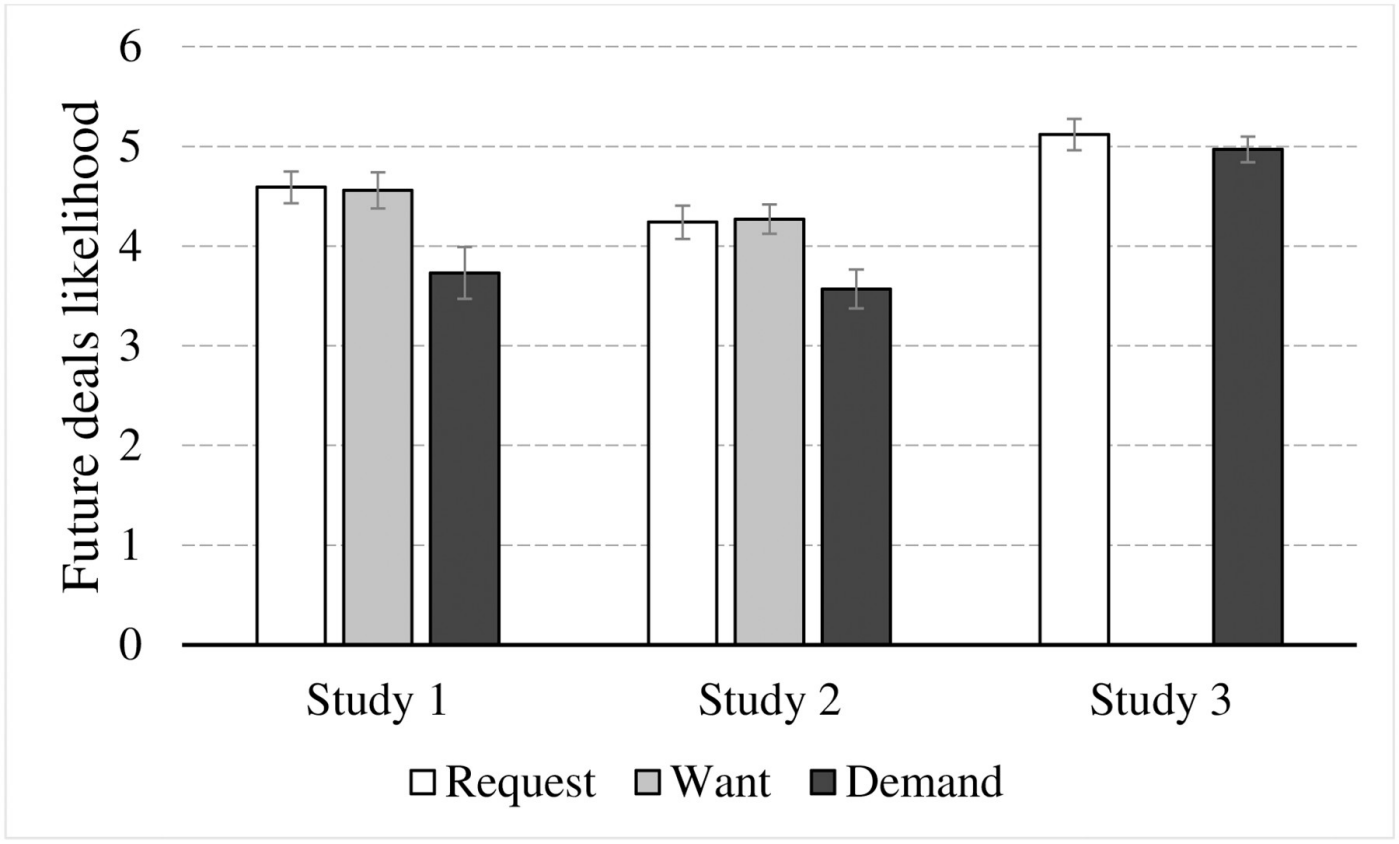
Averaged likelihood of conducting future deals as a function of the language used to request a discount in all three experiments. Vertical lines depict standard error.

To test H1b we conducted ANOVA with planned contrast to examine the effect of language extremity on the willingness to engage in future deals. The descriptive statistics for the analysis are presented in Fig 3 (left columns). As we predicted, language extremity had a significant effect on the willingness to engage in future deals (F(2, 122) = 5.579, p < 0.01, η_p_^2^ = .084). As can be seen in Fig 3, willingness to engage in future deals was higher for requesting and wanting (Mean = 4.59, SD = 1.0, 95% CL [4.18, 4.99], and Mean = 4.56%, SD = 1.2, 95% CL [4.16, 4.96], respectively) than for demanding a discount (Mean = 3.7, SD = 1.7, 95% CL [3.32, 4.14]). Planned contrast further revealed that willingness to engage in future deals was significantly different between request and demand (p < 0.005), and between want and demand (p < 0.005). No difference was found between request and want (p = 0.93). This pattern of results supports H1b.

**Fig 4:**
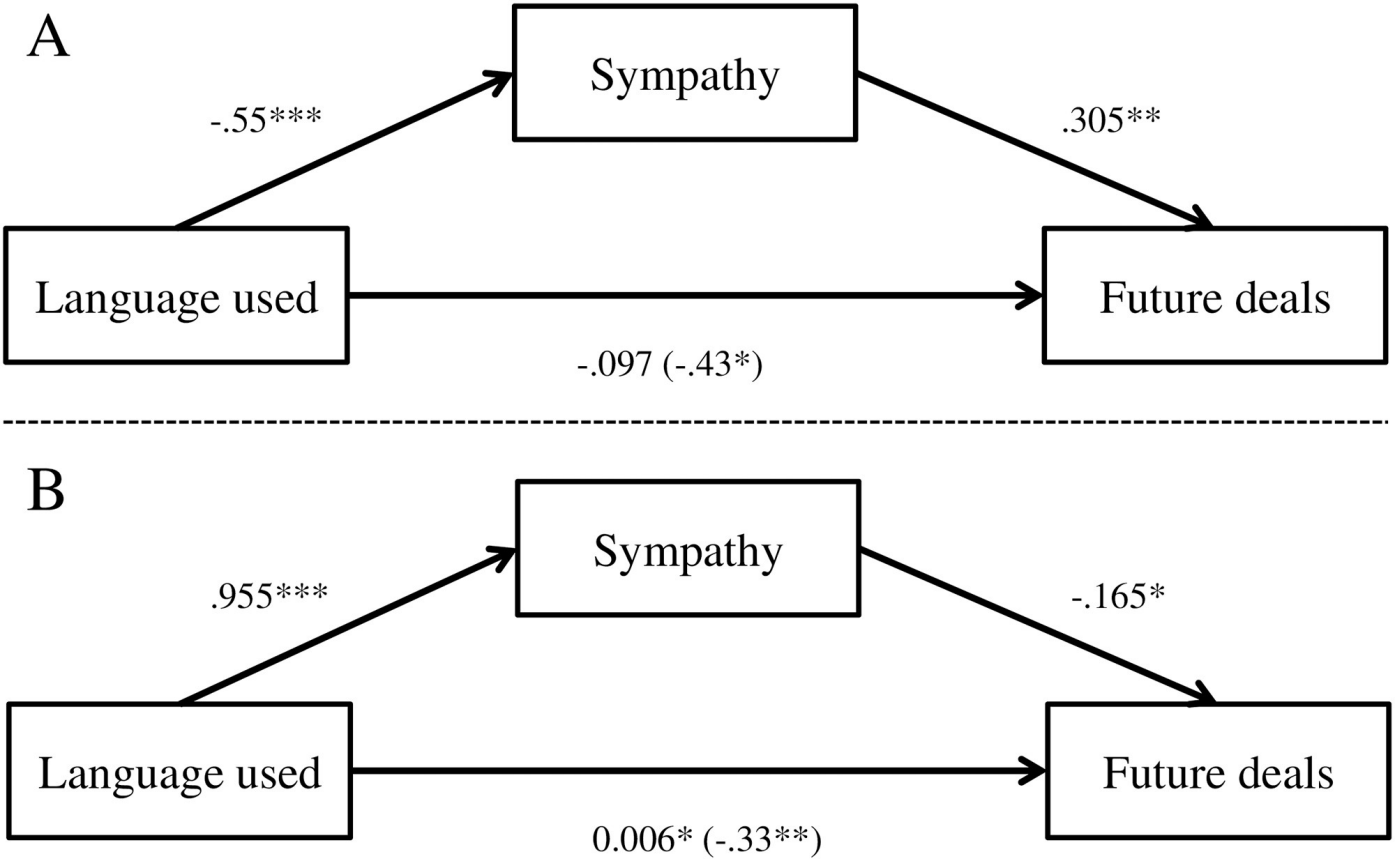
The effect of language used on likelihood of conducting future deals mediated by experienced emotions. All entries are raw (unstandardized) coefficients. The association between the mediator and the DV is represented by a coefficient from a model where the IV is also a predictor of the DV. Numbers in parentheses refer to the direct effect of the language used on likelihood of conducting future deals. Panel A: Study 1. Panel B: Study 2. * < 0.05, ** < 0.01, *** < 0.0001.

To test H3b, and H4b, we conducted mediation analysis using the PROCESS macro (PROCESS Model 4; [80]) in SPSS v21 with bias-corrected bootstrapped confidence intervals (CI). Language extremity was negatively related to empathy and sympathy and positively correlated with anger (see discount rate results). In addition, in line with the ANOVA results, language extremity was negatively related to willingness to conduct future deals (β = -.43, SE = 0.15, 95% CL [-.717, -.136], p < 0.05; R^2^_Adj_ = 6.4%). However, when empathy, sympathy and anger were entered to the model as mediators, language extremity no longer had a significant relationship with willingness to engage in future deals (β = -.097, SE = 0.16, 95% CL [-.412, .218], p = 0.54), and only sympathy (β = .305 SE = 0.13, 95% CL [.050, .560], p < 0.01) remained significant (F(4, 120) = 7.01, p < 0.0001; R^2^_Adj_ = 18.9%). Thus, just like with discount rate, H3b was not supported, and a mediation effect (H4b) was found for sympathy alone. The mediation results are illustrated in Fig 4 (Panel A).

The results of Study 1 provided initial support for the hypotheses that requesting a discount (versus demanding a discount) would lead to the highest discount rate and a greater willingness to engage in future deals. Furthermore, sympathy was found to mediate between the linguistic cue and the negotiation outcomes, such that the positive emotion increased discount rates and the willingness to engage in future deals. However, due to the culturally ordained nature of language, we wanted to expand the research to English speakers and examine whether native English speakers would respond similarly to native Hebrew speakers.

## Study 2

### Materials and methods

#### Ethics statement

The study and consent procedure were reviewed and approved by the Interdisciplinary Center’s School of Psychology Institutional Review Board before it was administered. In accordance with the ethics protocol, participants provided electronic consent for participation at the beginning of the study.

#### Participants

One-hundred twenty-eight undergraduate students (44 male) from a private university in the center of Israel participated in the experiment. It should be noted that data was collected in two separate stages–two consecutive semesters in the same course–and was aggregated for data analysis. All participants were international students, who attended a course on language and thought. Thus, sample size was not determined a-priori. Their average age was 23.5 years (SD = 4.2). Participation was voluntary and conducted upon consent at the end of class. Participants were randomly assigned to one of three between-subject conditions.

#### Design and procedure

The design and procedure were identical to Study 1, with the exception that all vignettes were in English.

As in Study 1, in addition to the main study a separate pretest of native English speakers (n = 42) was conducted to verify whether the words ‘request’, ‘want’, or ‘demand’ (in English) represent different levels of language extremity. Participants were undergraduate students from the same private university who attended a different course. They were asked to indicate to what extent, ‘I request, ‘I want’ and ‘I demand’ (in a random order) represent a soft or tough appeal, on a 10-point scale (1 = very soft, and 10 = very tough).

### Results and discussion

#### Manipulation check

The average rating of soft-tough was highest for ‘I demand’ (Mean = 8.76, SD = 2.18), moderate for ‘I want (Mean = 6.10, SD = 2.05), and lowest for ‘I request’ (Mean = 3.79, SD = 2.32). Repeated-measures ANOVA revealed that the language used had a significant effect on participants’ ratings (F(2, 82) = 46.251, p < 0.0001, η_p_^2^ = .53). In addition, planned contrast further revealed a significant difference between ask and want (t(41) = -4.682, p < 0.0001), want and demand (t(41) = -5.96, p < 0.0001), and ask and demand (t(41) = -8.287, p < 0.0001). This pattern of results suggests that in English as well, the language used affects perceived language extremity, and that request leads to the highest level, while ask leads to the lowest level of extremity.

#### Discount rate

To test H1a we conducted ANOVA with planned contrast to examine the effect of language extremity on discount rate. The descriptive statistics for the analysis are presented in Fig 1 (middle columns). As we predicted, language extremity had a marginally significant effect on discount rate (F(2, 125) = 2.761, p = 0.07, η_p_^2^ = .042). As can be seen in Fig 1, the discount rate was higher for requesting and wanting (Mean = 10.7%, SD = 9.0, 95% CL [8.54, 12.85], and Mean = 9.9%, SD = 6.7, 95% CL [7.78, 11.99], respectively) than for demanding a discount (Mean = 7.2%, SD = 4.7, 95% CL [5.08, 9.39]). Planned contrast further revealed that discount rate was significantly different between request and demand (p < 0.05), and marginally significant between want and demand (p = 0.08). No difference was found between request and want (p = 0.60). This pattern of results supports H1a.

Next, we tested H2, H3a, and H4a. Since our manipulation check results suggested that in English too, the language used falls on an extremity of language continuum, we conducted mediation analysis using the PROCESS macro (PROCESS Model 4; [80]) in SPSS v21 with bias-corrected bootstrapped confidence intervals (CI). The results revealed that language extremity was negatively related to empathy (β = -.667, SE = 0.12, 95% CL [-.906, -.428], p < 0.0001; R^2^_Adj_ = 19.5%) and sympathy (β = -.60, SE = 0.13, 95% CL [-.857, -.334], p < 0.0001; R^2^_Adj_ = 13.8%), and positively correlated with anger (β = .60, SE = 0.12, 95% CL [.352, .839], p < 0.0001; R^2^_Adj_ = 15.7%). Thus, in support of H2, language extremity was negatively correlated with positive emotions and positively correlated with negative emotions.

In addition, in line with the ANOVA results, language extremity was negatively related to discount rate (β = -1.73, SE = 0.77, 95% CL [-3.247, -.206], p < 0.05; R^2^_Adj_ = 3.9%). However, when empathy, sympathy and anger were entered to the model as mediators, language extremity no longer had a significant relationship with discount rate (β = -.275, SE = 0.86, 95% CL [-1.970, 1.420], p = 0.75), and only empathy (β = 1.842 SE = 0.63, 95% CL [.589, 3.094], p < 0.005) remained significant (F(4, 123) = 4.756, p < 0.005; R^2^_Adj_ = 13.4%). Thus, H3a was not supported. However, the pattern of results is consistent with a mediation effect (H4a) for empathy alone (and not sympathy as we found in Study 1. We refer to this in the discussion section). The mediation results are illustrated in Fig 2 (Panel B).

#### Future deals

To test H1b, we conducted ANOVA with planned contrast to examine the effect of language extremity on the willingness to engage in future deals. The descriptive statistics for the analysis are presented in Fig 3 (middle columns). As we predicted, language extremity had a significant effect on the willingness to engage in future deals (F(2, 125) = 5.353, p < 0.01, η_p_^2^ = .079). As can be seen in Fig 3, willingness to engage in future deals was higher for requesting and wanting (Mean = 4.24, SD = 1.1, 95% CL [3.90, 4.58], and Mean = 4.27%, SD = 1.0, 95% CL [3.94, 4.61], respectively) than for demanding a discount (Mean = 3.6, SD = 1.3, 95% CL [3.23, 3.91]). Planned contrast further revealed that willingness to engage in future deals was significantly different between request and demand (p < 0.01), and between want and demand (p < 0.005). No difference was found between request and want (p = 0.89). This pattern of results supports H1b.

To test H3b and H4b, we conducted mediation analysis using the PROCESS macro (PROCESS Model 4; [80]) in SPSS v21 with bias-corrected bootstrapped confidence intervals (CI). Language extremity was negatively related to empathy and sympathy and positively correlated with anger (see discount rate results). In addition, in line with the ANOVA results, language extremity was negatively related to willingness to conduct future deals (β = -.33, SE = 0.12, 95% CL [-.576, -.091], p < 0.01; R^2^_Adj_ = 5.6%). However, when empathy, sympathy and anger were entered to the model as mediators, language extremity no longer had a significant relationship with willingness to engage in future deals (β = .006, SE = 0.13, 95% CL [-.250, .263], p = 0.54), while sympathy (β = -.165 SE = 0.09, 95% CL [-.334, .005], p = 0.06) and anger (β = .256 SE = 0.09, 95% CL [.084, .428], p = 0.06) remained significant (F(4, 120) = 9.22, p < 0.0001; R^2^_Adj_ = 23.0%). Thus, H3b and H4b were supported, and a mediation effect (H4b) was found for sympathy and anger. The mediation results are illustrated in Fig 4 (Panel B).

The results of Study 2, similar to Study 1, demonstrated that requesting a discount (versus demanding a discount) would lead to the highest discount rate and a greater willingness to engage in future deals. Furthermore, positive emotions mediated between the linguistic cue and the negotiation outcome, such that positive emotions increased discount rates and the willingness to engage in future deals and negative emotions did the opposite to that. The findings revealed that native English and native Hebrew speakers responded to linguistic cues in the context of negotiations in a similar way, although the mediating emotions were different. Considering the latter findings and the difference in native and foreign language use in eliciting emotional reactions [81], we wanted to examine whether using a foreign language would elicit similar emotional intensity as found in the two previous studies, in which the speakers used their native language (whether Hebrew or English), and whether this emotional reaction would mediate the influence of language extremity on negotiation outcomes.

## Study 3

### Materials and methods

#### Ethics statement

The study and consent procedure were reviewed and approved by the Interdisciplinary Center’s School of Psychology Institutional Review Board before it was administered. In accordance with the ethics protocol, participants provided electronic consent for participation at the beginning of the study.

#### Participants

Eighty-one Israelis (33 male) participated in the experiment. Participants were recruited using Midgam panel service (www.midgam.com) in an attempt to examine the robustness of our findings among a non-student population. All participants were native Hebrew speakers, and their average age was 43.7 years (SD = 16.2). To be consistent with the previous studies, sample-size was not determined a-priori. Participation was voluntary and conducted upon consent. Participants were compensated 2USD as participation fee. The participants were randomly assigned to one of two between-subject conditions.

#### Design and procedure

The design and procedure were identical to Studies 1 and 2 with the exception that the customers’ responses were presented in English to Hebrew speaking participants. In addition, since the results of Study 1 and 2 showed no difference between requesting and wanting, and requesting and demanding represent the opposite ends of the extremity continuum, Study 3 included only these two linguistic cue conditions.

### Results

#### Discount rate

To test H1a, we conducted ANOVA to examine the effect of language extremity on discount rate. The descriptive statistics for the analysis are presented in Fig 1 (right-hand columns). As we predicted, language extremity had a significant effect on discount rate (F(1, 78) = 3.845, p = 0.05, η_p_^2^ = .047). As can be seen in Fig 1, the discount rate was higher for requesting (Mean = 8.8%, SD = 3.6, 95% CL [7.65, 10.00]) than for demanding a discount (Mean = 7.2%, SD = 3.8, 95% CL [6.06, 8.35]). As in Studies 1 and 2, this pattern of results supports H1a.

To test H2, H3a, and H4a, we conducted mediation analysis using PROCESS macro (PROCESS Model 4; [80]) in SPSS v21 with bias-corrected bootstrapped confidence intervals (CI). The results revealed that language extremity was negatively related to empathy (β = -.702, SE = 0.28, 95% CL [-1.250, -.155], p < 0.05; R^2^Adj = 7.7%), and positively correlated with anger (β = .948, SE = 0.24, 95% CL [.476, 1.420], p < 0.0001; R^2^Adj = 17.0%). Thus, in support of H2, language extremity was negatively correlated with positive emotions (though not with sympathy) and positively correlated with negative emotions.

In addition, in line with the ANOVA results, language extremity was negatively related to discount rate (β = -1.61, SE = 0.82, 95% CL [-3.251, .025], p = 0.06; R^2^_Adj_ = 4.7%). When empathy, sympathy and anger were entered to the model as mediators, language extremity no longer had a significant relationship with discount rate (β = -.731, SE = 0.90, 95% CL [-2.518, 1.054], p = 0.42). Similarly, anger (p = 0.18), empathy (p = 0.55), and sympathy (p = 0.47) did not remained significant. Thus, H3a and H4a were not supported.

#### Future deals

To test H1b we conducted ANOVA to examine the effect of language extremity on the willingness to engage in future deals. The descriptive statistics for the analysis are presented in Fig 3 (right-hand columns). Contrary to H1, language extremity had no effect on the willingness to engage in future deals (F(1, 78) = 0.52, p = 0.47, η_p_^2^ = .007). As can be seen in Fig 3, the likelihood to engage in future deals was slightly lower for requesting (Mean = 4.97, SD = 1.0, 95% CL [4.68, 5.26]) than for demanding a discount (Mean = 5.1, SD = 3.8, 95% CL [4.84, 5.41]). Thus, unlike in Study 1 and 2, H1b was not supported, suggesting that foreign language had a weaker effect on behavior relative to native language [82].

To test H3b, and H4b, we conducted mediation analysis using the PROCESS macro (PROCESS Model 4; [80]) in SPSS v21 with bias-corrected bootstrapped confidence intervals (CI). Language extremity was negatively related to empathy and sympathy and positively correlated with anger (see discount rate results). However, as willingness to engage in future deals was not related to language extremity (β = .15, SE = 0.20, 95% CL [-.260, .555], p = 0.47), we found no support for H3b and H4b.

## Conclusions

Psycholinguistic research on the power of language suggests that language has the power to shape emotions [10] within a persuasion framework [11] as well as shaping negotiating preferences [12]. Building on this line of research, in the current work we explored an extremely subtle means of inducing preferences in the context of negotiations. Specifically, we examined whether subtle differences in asking for a discount (request, want, or demand) in the context of negotiations would induce different levels of emotions and in turn, different outcomes. Results across three experimental studies supported this prediction for the most part of: language extremity influenced the negotiators’ emotions, which in turn led to significant changes in the negotiation outcomes. Linguistic cues influenced negotiation outcomes, such that requesting or asking for a discount led to higher discount rate and willingness to engage in future deals relative to demanding. It is important to note that initially we assumed that linguistic extremity would have a linear effect. However, interestingly, this is not the case. It appears that there is a “threshold effect” in which only when the language becomes extreme enough, a threshold is crossed and consequently our emotions and decisions are altered. In addition, the negotiator’s emotions mediated between linguistic cues and the negotiation outcome, such that positive emotions mediated this effect, but only when the linguistic cues were portrayed in the responders’ native tongue.

The current research is an important first step in demonstrating that language extremity plays an important role in negotiations. Therefore, it is important to first demonstrate that when all else is neutralized, the extremity of the language has a direct effect on the other side’s experienced emotions, which in turn affect their behavioral tendencies.

### Psychological mechanisms of language, emotions and negotiations

Understanding how emotions mediate the connection between language and negotiation outcomes sheds light on the psychological mechanism behind negotiation dynamics. The current research accentuates the role of emotions in the negotiators’ decisions, and calling negotiation scholars to focus on the subject. The fact that such subtle changes in language resulted in major differences in emotions, attitude judgments and behavior suggests that negotiation research may indeed benefit from adopting a persuasion framework [21].

Past research in neuro-economics suggests that one’s neurophysiological processes are both emotional (i.e., arousal) and cognitive (i.e., beliefs, assumptions and perceptions), involving a bidirectional link alongside environmental factors that may affect these processes. The latter internal neurophysiological processes lead the individual to develop her/his own assessment of the current relevant risks and benefits, which in turn influence her/his behavior in the immediate situation [83]. In the current research, different words used to verbalize a desired goal served as environmental cues, which in turn triggered certain emotions (e.g. sympathy in Study 1 in response to the use of the word “ask” and “request”).

### Implications

As all three studies revealed, the use of a less extreme language (‘request’ and ‘want’), led to significantly higher discount rates and willingness to engage in future deals than a more extreme language. This correlates with previous persuasion literature [11,84] regarding the influence of used language on ’real world’ decisions and behavior. Seeing how common and prominent negotiation is in our day-to-day life, our results may have some substantial implications. Negotiation oriented situations can vary from an important executive meeting regarding the future of an employee’s career to a vigorous family discussion revolving around the desired bedtime of the children.

During negotiation, both parties tend to focus on the desired outcomes of the event, and therefore the terms of their demands [17]. Yet, the current research reveals that when putting actual demands aside, the language we use to convey them can create a meaningful difference in the partner’s willingness to comply with them. This information can actually benefit us twice; understanding the way emotions influence the outcomes of negotiation can guide us not only into choosing the right phrasing when we make a certain offer, but also to introspect our own decision-making process when receiving one. This introspection may lead us to make choices that truly reflect our future goals rather than our current emotional state [85]. Our emotional framework is a starting point, not a culmination. Depending on how we approach emotions in negotiation, we may be either slaves or masters to them—with varying consequences [65]. Indeed, research in the field of emotional regulation teaches us that awareness of changes occurring in our emotional state has the potential to lead to the application of corrective processes to our evaluative judgments [85].

Interestingly, while previous research in social psychology suggests that negative emotions have a stronger effect on behavior than positive emotions (see e.g., [59,60]), negotiation scholars have demonstrated the benefits of both positive [55] and negative emotions [13]. While attempts have been made to understand the complex influence of different emotions alongside the various negotiation settings, mediators and moderators [86], more research is needed to draw the full picture. In this sense, our results contribute to the research in the field by pointing to a specific situation where positive emotions are more beneficial than negative ones. Future research can further explore such cases and their theoretical explanations [87]. Moreover, the effect of emotional awareness leaves the gate open for further inquiry; will letting people self-reflect on the emotions rising within them, as responses to different phrasing of asking, lead to a milder affect measured in given discount rates?

Another aspect of the current results is the extent to which linguistic cues affected the way people perceived their future actions. People were more willing to engage in future deals when the linguistic cues signified a request rather than a demand. Many times, a negotiation situation is just one part of an established long-term relationship, of a financial or even social kind. When one approaches this type of negotiation, regardless to whether specific demands are answered or not, it is of significance to the continuation of the relationship. Understanding how emotions affect willingness to engage in future interactions, may help us behave in a way that will enhance future likelihood for those to occur.

### Sympathy as mediator between the linguistic cue and the negotiation outcome

A significant finding of the current research is the effect the linguistic cue (request) had on sympathy and the latter’s contributing role to the outcome of the negotiation. This finding accentuates the impact of requesting (versus demanding) on inducing sympathy and, additionally, the significant role sympathy has as mediator between the request and the positive outcome. In light of the current finding, it is important to understand the nature of sympathy in the context of negotiations.

The question of what prompts human sympathy has been of great importance to humanity, and although it does not operate according to any kind of normative rules or higher-level principles, it is impacted indirectly by calculations of deservingness and is affected by several situations and stimuli [88]. First, one’s personal state affects the level of sympathy, specifically the relation between one’s feeling and that of the target individual (in our case, the partner involved in the negotiation). Second, past and vicarious experience has been found to increase sympathy, empathy and the likelihood of assisting (in our case, the willingness to compromise) [89]. Finally, novelty has been found to induce sympathy, although humans are adaptive in nature and with time may become less sympathetic to a given target [90]. Within a negotiation framework, a first encounter is novel.

It is important to note that research on sympathy has focused on public issues and situations identifiable with victim effect. The current research suggests that sympathy is a prominent factor in determining the outcome of a negotiation, and proposes that language is a contributing factor in evoking sympathy within this framework. Our findings are suggestive, but certainly call for extended studies on the role of language, sympathy and negotiation contexts.

### Native versus foreign language use and level of emotionality in the negotiation process

The results of the current studies reveal a significant difference between native and foreign language use regarding emotions as mediators. In Study 1, sympathy mediated between the linguistic cue and the negotiation outcomes, such that the positive emotion increased discount rates and the willingness to engage in future deals. In Study 2, empathy mediated between the linguistic cue and the negotiation outcome, such that positive emotions increased discount rates and the willingness to engage in future deals and negative emotions did the opposite to that. The findings revealed that native English and native Hebrew speakers responded to linguistic cues in the context of negotiations in a similar way, although the mediating emotion was different. Research has suggested that foreign teachers are more empathetic towards their students [91]. In line with this finding, the foreign participants in Study 2 may have been more inclined to be empathetic as they were in a situation requiring familiarity and use of norms of negotiation in the local setting (which they are less likely to have). This could be examined in future research.

However, in Study 3, emotions did not mediate between the linguistic cue and the negotiation outcome, suggesting that using a foreign language does not elicit the same level of emotional intensity [81] and behavior [92] as using one’s native language. These results are significant for negotiations carried out within a foreign setting, which requires negotiators to use a language that is foreign to them. The latter possibly reduces emotional intensity, which in turn, leads to more practical and functional decision-making processes, or as Costa et al. [92] suggest, an increase in utilitarian judgments.

### The role of linguistic cues in the anchoring effect

Given the importance and centrality of first offers in negotiation research in the past few decades, the current work highlights the advantages of focusing on emotional and behavioral responses to first offers and their effects on the negotiation process. Negotiation literature suggests that the first offer in a negotiation serves as an anchor that influences both the counteroffer and the settlement price. According to the anchoring and adjustment heuristic [93], when people try to estimate an unknown quantity (for instance, an opponent’s reservation price in a negotiation), they tend to anchor on a given number (e.g., the first offer in a negotiation) and adjust from it. Findings propose that this anchoring influences both experienced and novice negotiators in biasing the final decision toward the initial value [94]. Given the robustness of the anchoring effect, studies have concluded that an anchoring tactic of making an extreme but reasonable first offer is effective in maximizing profits in negotiations [1,21]. Our findings suggest that one of the ways to react to a first offer is not by presenting a counter offer, but rather by requesting a discount. In line with Kahneman’s [95] and Trötschel et al. [17] studies, the way a proposal is framed in a negotiation process may influence the negotiating parties’ concession aversion and their willingness to concede to the other party’s proposal. The current study serves as a first step in researching the request for a discount as a strategy for overcoming the anchoring effect of the first offer. Should further research investigate this, the wording of the first offer must be taken into consideration.

### Limitations

The findings of the current studies should be considered with some limitations. First, in the current research, a computer-based negotiation was carried out. This approach was used in order to investigate linguistic cues without letting any other factors influence their effects (e.g. body language). However, in studying negotiations, live simulations may increase the reliability and validity of the findings, especially when emotions comprise a significant role in the process. Additionally, the emotions were measured using a self-report scale. An in-depth account given by the negotiators regarding the emotions that were induced during the process may, similar to the first point, increase the reliability and validity of the findings. Furthermore, in the current study the negotiation process was somewhat "abridged" and a complete process from the very beginning to the very end may provide a more realistic construal of the experience. Similar to previous research [14, 15], we used the first step in the negotiation process, rather than examining an interactive process. This was done to isolate the direct effect of language extremity. Still, future research should examine the effect of language extremity in more interactive processes.

Additionally, it is important to note that in the experimental negotiation context involving buyers and sellers, the buyer does not have the possibility of choosing another seller. This may place the buyer in a lower position (one-down; [96]), resulting in a sensitivity to linguistic and emotional cues. Past research in negotiation that has examined first offers and counteroffers has generally demonstrated symmetric effects on buyers and sellers without any role effect (e.g. [2, 5]).

Moreover, although the three studies support our hypotheses for the most part, it is important to note that the relatively small effect size requires further research to include a larger sample in order to achieve greater precision and power. Finally, our extremity manipulation included not only different wording, but also different punctuation marks. For example, the request was followed by a question mark, while the demand was followed by an exclamation mark. Thus, one might argue that the different marks affected the results, and not the different wording. However, while the different marks are universal, wording is specific to one’s native language. The reduced effect found for wording in a foreign language (Study 3), suggests that our results are predominated by the wording and not the marks.

### Future research

In line with the above limitations, future research might also explore live negotiations and live measurement of emotional and cognitive processes during the interaction. While we only examined discount rate and willingness to engage in future deals, we believe our results extend beyond these two measures and represent a more general behavioral tendency. Of course, this assumption should be examined in future research. Additionally, it may be interesting to examine the nature and valence of emotions when the negotiating partners are acquaintances. Specifically, are different mechanisms at play when we are negotiating with acquaintances as opposed to business associates? Finally, will raising people’s awareness of their changing emotional state during negotiation result in different outcomes? If language helps to constitute emotions in the first place by shaping how people make meaning of affective states, then it follows that prompting people to make meaning of their states with linguistic categories (as in affect labeling) or to re-construe the meaning of an emotion with a different linguistic category (as in reappraisal) will contribute to the regulation of emotions [97].
